# MRSA carrying *mecC* in captive mara

**DOI:** 10.1093/jac/dkv024

**Published:** 2015-02-25

**Authors:** C. Espinosa-Gongora, E. M. Harrison, A. Moodley, L. Guardabassi, M. A. Holmes

**Affiliations:** 1 Department of Veterinary Disease Biology, Faculty of Health and Medical Sciences, University of Copenhagen, Stigbøjlen 4, 1870 Frederiksberg C, Denmark; 2 Department of Veterinary Medicine, University of Cambridge, Madingley Road, Cambridge CB3 0ES, UK

**Keywords:** methicillin resistance, wildlife, zoo, WGS

## Abstract

**Objectives:**

To characterize the staphylococcal cassette chromosome *mec* (SCC*mec*), virulence and antimicrobial susceptibility of *Staphylococcus aureus* ST130 isolated from mara (*Dolichotis patagonum*), a large rodent species native to South America and kept in captivity at Copenhagen Zoo.

**Methods:**

The presence of *mecC* was confirmed by PCR in 15 *S. aureus* ST130 isolated from mara during a previous study. WGS was performed on two randomly selected isolates to characterize their genomes with respect to SCC*mec*, virulence and resistance gene content. Antimicrobial susceptibility was tested using commercial broth microdilution tests.

**Results:**

All the isolates belonged to *spa* type t528 ST130 and carried *mecC*. Based on WGS, *mecC* was 100% identical to the prototype sequence of *S. aureus* strain LGA251. The sequence of SCC*mec* type XI in the mara isolates had 23 SNPs compared with the one described in LGA251. The two sequenced strains harboured a set of virulence factors and other genomic features previously observed in ST130. Both strains carried *norA* as the only putative antimicrobial resistance gene in addition to *mecC*.

**Conclusions:**

Our findings support the notion that a genetically conserved *mecC*-carrying MRSA ST130 clone is widespread in a variety of unrelated hosts in Denmark. Since the mara at Copenhagen Zoo have limited contact with humans and other animal species, it remains unclear whether mara are natural hosts of ST130 or acquired this lineage from unknown sources. The broad host range of MRSA ST130 supports its designation as a generalist lineage.

## Introduction

*Staphylococcus aureus* carrying *mecC* is a novel MRSA variant present in Europe, largely associated with the clonal lineage CC130 (and associated STs) and with ST425. It was first isolated from bulk tank milk and humans,^1^ and subsequently from cattle,^2^ sheep,^3^ domestic dogs, cats and guinea pigs^4,5^ and a broad range of wild animal species including seals, chaffinch, rats, rabbits,^6^ hare, otters,^7^ hedgehogs^7,8^ and wood mice.^9^ In Denmark, the number of *mecC*-positive MRSA cases in humans (including colonization and infection) was 9 in 2009, 21 in 2010, 37 in 2011 and 24 in 2012. Among the 24 cases reported in 2012, 16 (67%) were infections, including one case of bacteraemia. Contact with livestock was not registered in any of the cases.^10^ However, transmission of *mecC*-positive MRSA between livestock and humans has been previously demonstrated by WGS in two cases in Denmark.^11^

This study reports 15 isolates of *mecC*-positive MRSA isolated from 15 captive mara (*Dolichotis patagonum*), a large rodent species native to South America. The isolates were obtained during a study on the population structure of *S. aureus* at Copenhagen Zoo in 2010.^12^ In that study, seven *S. aureus* lineages were found among 25 mammalian species, with two STs (ST130 and ST133) being isolated from mara. All isolates were negative for *mecA* by PCR and did not grow on Brilliance MRSA Agar (Oxoid, UK). These isolates had a typical *mecC* MRSA phenotype, shown by their susceptibility to oxacillin, low cefoxitin MICs (8 mg/L) and failure to grow on commercial selective agars.^12,13^ In the present study, the isolates were further investigated to characterize the staphylococcal cassette chromosome *mec* (SCC*mec*), virulence determinants and antimicrobial susceptibility.

## Materials and methods

Fifteen *S. aureus* isolates from mara (comprising the seven isolates described in the original paper^12^ together with a further eight isolates obtained from nasal swabs taken from the same group of animals) were subjected to *spa* typing and MLST as previously described.^12^ ST130 isolates were screened for the presence of *mecC* by PCR. WGS was performed on two randomly selected *mecC-*positive isolates designated Mara 1 (Z_37) and Mara 2 (Z_38). The SCC*mec* sequence and the presence of virulence and resistance genes were analysed using the Artemis Comparison Tool (ACT)^14^ and BLAST.^15^ Antimicrobial susceptibility was tested for a range of antibiotics, including amikacin, amoxicillin/clavulanic acid, ampicillin, cefazolin, cefovecin, cefoxitin, cefpodoxime, chloramphenicol, clindamycin, doxycycline, enrofloxacin, erythromycin, gentamicin, marbofloxacin, oxacillin, penicillin, rifampicin and trimethoprim/sulfamethoxazole, by broth microdilution using commercial custom-made MIC plates (Sensititre, TREK diagnostics, Cleveland, OH, USA). Results were interpreted based on the current CLSI breakpoints.^16^ Genomic DNA of *S. aureus* isolates Mara 1 (Z_37) and Mara 2 (Z_38) was extracted from overnight cultures grown in tryptic soy broth (TSB) at 37°C using the MasterPure Gram Positive DNA Purification Kit (Cambio, Cambridge, UK). Illumina library preparation was carried out as described by Quail *et al.*^17^ and Hi-Seq sequencing was carried out following the manufacturer's standard protocols (Illumina, Inc., San Diego, CA, USA). Genomes were assembled *de novo* from Fastqs with Velvet.^18^ The draft sequences for Mara 1 (Z_37) and Mara 2 (Z_38) had a total of 18 and 21 contigs, respectively. Comparative genomics was carried out using WebACT^19^ and viewed with the ACT.^14^ The presence of antibiotic resistance genes was identified using the ResFinder-1.3 Server (http://cge.cbs.dtu.dk/services/ResFinder/) and by BLAST against the assemblies. The presence of *S. aureus* virulence factors was identified by BLAST against the assemblies. Nucleotide sequences of isolates Mara 1 (Z_37) and Mara 2 (Z_38) have been deposited in the European short read archive with accession numbers ERR294351 and ERR294349, respectively. The assemblies used in this study are available on request to the corresponding author.

## Results and discussion

The 15 ST130 isolates from mara harboured *mecC* and belonged to *spa* type t528, which has previously been isolated from skin superficial infections (*n* = 4), wounds (*n* = 3) and blood (*n* = 2) infections in Danish patients.^2^ The *mecC* sequences were 100% identical to that described in the prototype *S. aureus* strain LGA251. Both mara strains carried SCC*mec* type XI with 23 SNPs compared with the prototype SCC*mec* type XI in LGA251. This SCC*mec* type has been found associated with *mecC* in various STs within CC130 as well as in other clonal complexes, such as CC49, CC425, CC599 and CC1943/6,^20^ indicating multiple acquisition events by distinct *S. aureus* lineages.

The following genes encoding virulence factors were detected: *hla*, *hlb*, *hlgACB*, *lukED*, *eta*, *edin-B*, *set2*, *set3*, *set4*, *set5*, *set7*, *set10* and a previously described variant of *etd* (*etd2*). The 3.3 kb deletion of the collagen adhesin gene (*cna*) was also found in the isolates from mara. Apart from *mecC* and *blaZ* as part of SCCmec XI, a 2.3 kb deletion of an EamA-like transporter family gene was also present in the mara strains. The similar set of virulence factors, resistance determinants and other genomic features of the mara strains compared with previously sequenced *mecC*-positive MRSA ST130 strains isolated from humans, sheep and cows in Denmark^11^ suggests a conserved genome within this lineage and clonal spread within the country. All sequenced Danish strains also carried the same genes for exfoliative toxins (*eta* and the *etd* homologue *etd2*), α- β- and γ-haemolysins (*hla*, *hlb* and *hlgABC*), epidermal cell differentiation inhibitor-B (*edin-B*), leucocidin ED (*lukED*) and superantigen-like proteins (*set2*, *set3*, *set4*, *set5*, *set7* and *set10*). Exfoliative toxins encoded by genes such as *eta* and *etd* are responsible for the loss of adherence between keratinocytes leading to damage of specific host tissues^21,22^ and, together with the epidermal cell differentiation inhibitor-B, haemolysins and leucocidin ED, influence the virulence potential of *S. aureus* strains. Given the high species specificity of exfoliative toxins,^23^ it has been suggested that the presence of the *etd* homologue *etd2* in *mecC*-positive MRSA^8,9,11,24^ may indicate an evolutionary step towards host adaptation.^11^

All the isolates showed resistance to ampicillin (MIC ≥0.5 mg/L), penicillin (MIC ≥0.5 mg/L) and cefoxitin (MIC ≥8 mg/L), but were susceptible to oxacillin (MIC <0.25 mg/L), according to current CLSI breakpoints for MRSA detection. Strains were susceptible to the remaining antimicrobials included in the test. This phenotype corresponds to the previously described biochemical properties of the *mecC*-encoded PBP2a, which confers higher resistance to cefoxitin than oxacillin due to a higher affinity to the latter antimicrobial.^25^ In addition to *mecC* and *blaZ* (as part of the *mec* complex class E), the two sequenced isolates also carried *norA*, which encodes an efflux pump able to transport fluoroquinolones, biocides and dyes. This putative resistance gene is known to confer resistance to these agents only when it is overexpressed.^26^

The finding of *mecC*-carrying MRSA ST130 in captive mara provides further evidence of the broad host range of this MRSA lineage. Based on previous reports, its apparent ability to colonize unrelated hosts, such as humans, rodents, ungulates, carnivores, birds and humans,^1–9^ raises questions about the origins of this lineage. The origin of *mecC*-carrying MRSA ST130 in mara at Copenhagen Zoo is unknown. Visitors to the zoo do not have direct contact with the mara and therefore any possible transmission route is likely to involve either zoo personnel with direct access to the animals or wildlife such as birds and small rodents, both of which have been previously reported to carry *mecC*-MRSA.^6,9^ Due to the conditions of captivity, it cannot be stated that mara are natural hosts of ST130; however, this was the only host species colonized with this lineage among the 25 mammalian species included in the previous study at the zoo.^12^ Furthermore, the fact that MRSA ST130 has found its way into a niche within the urban area of Copenhagen indicates that the spread of the lineage is not restricted to rural areas. This observation supports the notion that *mecC*-MRSA is spreading to new niches previously suggested in a recent study reporting *mecC*-MRSA in urban wastewater.^27^ It has been suggested that MRSA ST130 represents a new livestock-associated (LA) MRSA lineage based on its frequent isolation from bulk tank milks and confirmed transmission between ruminants and people exposed to them.^11^ However, the epidemiology of this lineage appears to differ from that of other LA MRSA lineages, such as ST398. First, none of the 24 human cases registered in Denmark in 2012 reported any contact with livestock,^5^ supporting previous evidence that this lineage is able to transmit between humans.^28^ Second, the broad host spectrum, including a list of wild species with unknown contact with livestock, supports its previous designation as a *generalist lineage*.^11^ Other authors have also questioned the association of *mecC*-carrying MRSA ST130 with livestock based on the recent finding of human adaptation genes *sak* and *scn* in *mecC*-MRSA isolated from wild rodents in Spain.^9^

In conclusion, this study suggests the spread of a genetically conserved and potentially zoonotic *mecC*-carrying MRSA ST130 clone in Denmark, including urban areas. Further genomic research is needed to elucidate the origins of this lineage and to explain why it is able to adapt to a multitude of unrelated hosts.

## Funding

This study was supported by internal funding (C. E.-G., A. M. and L. G.) and by the Medical Research Council (E. M. H. and M. A. H.; G1001787/1).

## Transparency declarations

None to declare.
